# Study protocol of the TRICOLORE trial: a randomized phase III study of oxaliplatin-based chemotherapy versus combination chemotherapy with S-1, irinotecan, and bevacizumab as first-line therapy for metastatic colorectal cancer

**DOI:** 10.1186/s12885-015-1630-1

**Published:** 2015-09-09

**Authors:** Yoshito Komatsu, Chikashi Ishioka, Ken Shimada, Yasuhide Yamada, Makio Gamoh, Atsushi Sato, Tatsuro Yamaguchi, Satoshi Yuki, Satoshi Morita, Shin Takahashi, Rei Goto, Minoru Kurihara

**Affiliations:** 1 Department of Cancer Chemotherapy, Hokkaido University Hospital Cancer Center, Kita-15 Nishi-7 Kita-ku, Sapporo, 060-8638 Japan; 2 Department of Clinical Oncology, Institute of Development, Aging and Cancer, Tohoku University, 4-1 Seiryo-machi, Aoba-ku, Sendai, 980-8575 Japan; 3 Department of Internal Medicine, Showa University Kouto Toyosu Hospital, 5-1-38 Toyosu, Kouto-ku, Tokyo, 135-8577 Japan; 4 Gastrointestinal Medical Oncology Division, National Cancer Center Hospital, 5-1-1 Tsukiji, Chuo-ku, Tokyo, 104-0045 Japan; 5 Department of Medical Oncology, Osaki Citizen Hospital, 2-3-10 Senjujicho, Hurukawa, Osaki Miyagi 989-6183 Japan; 6 Department of Medical Oncology, Hirosaki University Graduate School of Medicine, 5 Zaifucho, Hirosaki, Aomori 036-8562 Japan; 7 Department of Surgery, Tokyo Metropolitan Cancer and Infectious diseases Komagome Hospital, 3-18-22 Honkomagome, Bunkyo-ku, Tokyo, 113-8677 Japan; 8 Department of Gastroenterology and Hepatology, Hokkaido University Hospital, Kita-15 Nishi-7 Kita-ku, Sapporo, 060-8638 Japan; 9 Department of Biomedical Statistics and Bioinformatics, Kyoto University Graduate School of Medicine, 54 Kawahara-cho, Shogoin, Sakyo-ku, Kyoto 606-8507 Japan; 10 Hakubi Center of Advanced Research, Kyoto University, Yoshida-Ushinomiyacho, Sakyo-ku, Kyoto 606-8501 Japan; 11 Graduate School of Economics, Kyoto University, Yoshida-Honmachi, Sakyo-ku, Kyoto 606-8501 Japan; 12 The Tokyo Cooperative Oncology Group, 2-1-18 Hamamatsu-cho, Minato-ku, Tokyo, 105-0013 Japan

## Abstract

**Background:**

Metastatic colorectal cancer carries a poor prognosis and cannot be cured by currently available therapy. Chemotherapy designed to prolong survival and improve the quality of life (QOL) of patients is the mainstay of treatment. Standard regimens of FOLFOX/bevacizumab and CapeOX/bevacizumab can cause neurotoxicity, potentially disrupting treatment. The results of 3 phase II studies of combination therapy with S-1, irinotecan, and bevacizumab showed comparable efficacy to mFOLFOX6/bevacizumab and CapeOX/bevacizumab, without severe neurotoxicity. Therefore, the establishment and evaluation of S-1-containing irinotecan-based regimens for first-line treatment are expected to become more important.

**Methods:**

The TRICOLORE trial is a multicenter, randomized, open-label, controlled phase III study which aims to evaluate the non-inferiority of combination therapy with S-1/irinotecan/bevacizumab (a 3-week regimen [SIRB] or 4-week regimen [IRIS/bevacizumab]) to oxaliplatin-based standard treatment (mFOLFOX6/bevacizumab or CapeOX/bevacizumab) in patients with metastatic colorectal cancer who had not previously received chemotherapy. Patients will be randomly assigned to either the control group (mFOLFOX6/bevacizumab or CapeOX/bevacizumab) or study group (SIRB or IRIS/bevacizumab). The target sample size is 450 patients. The primary endpoint is progression-free survival (PFS), and the secondary endpoints are overall survival (OS), response rate (RR), time to treatment failure (TTF), relative dose intensity (RDI), the incidence and severity of adverse events, quality of life (QOL), quality-adjusted life years (QALY), health care costs, and relations between biomarkers and treatment response (translational research, TR).

**Discussion:**

The results of this study will provide important information that will help to improve the therapeutic strategy for metastatic colorectal cancer, and we believe that this study is very meaningful from the perspective of comparative effectiveness research.

**Trial registration:**

UMIN000007834

**Electronic supplementary material:**

The online version of this article (doi:10.1186/s12885-015-1630-1) contains supplementary material, which is available to authorized users.

## Background

Metastatic colorectal cancer carries a poor prognosis and cannot be cured by currently available therapy. Chemotherapy designed to prolong survival and improve the quality of life (QOL) of patients is the mainstay of treatment. On the basis of the results of previous clinical studies [1–3], bevacizumab plus standard regimens such as FOLFOX, CapeOX, and FOLFIRI is currently recommended as first-line treatment for metastatic colorectal cancer.

In Japan, S-1, an oral fluoropyrimidine-based anticancer preparation, is approved for the indication of colorectal cancer. S-1 combines tegafur, a prodrug of 5-fluorouracil, with 2 modulators of 5-fluorouracil metabolism: gimeracil and oteracil potassium. Gimeracil reversibly inhibits dihydropyrimidine dehydrogenase (DPD), a catabolic enzyme of 5-fluorouracil, and thereby increases concentrations of 5-fluorouracil in serum and tumor tissue. Oteracil potassium suppresses the activity of 5-fluorouracil in gastrointestinal tissue, reducing gastrointestinal toxicity [4]. Many clinical trials have evaluated S-1 in combination with irinotecan. Komatsu et al. conducted phase I/II studies in gastric cancer and phase II studies in colorectal cancer [5, 6]. Subsequently, Muro et al. performed a Phase II/III study (FIRIS trial) comparing FOLFIRI with S-1/irinotecan (IRIS) as second-line therapy in patients with metastatic colorectal cancer. IRIS was demonstrated to be non-inferior to FOLFIRI in terms of progression-free survival (PFS). IRIS was thus established to be a viable option for the second-line treatment of metastatic colorectal cancer, similar to FOLFIRI [7].

The results of phase II studies evaluating S-1 plus irinotecan combined with bevacizumab as first-line treatment have been reported. In a study by Komatsu et al. [8], bevacizumab (5 mg/kg) and irinotecan (100 mg/m^2^) were given as a continuous intravenous infusion on days 1 and 15, and S-1 was given orally for 2 weeks, followed by a 2-week rest period. This 4-week regimen was regarded as 1 course. The RR was 57.7 % (95 % CI, 43.2 to 71.3 %), and the PFS was 16.7 months (95 % CI, 13.1 to 18.7 months). In a study performed by Yamada et al. [9], bevacizumab (7.5 mg/kg) and irinotecan (150 mg/m^2^) were given as a continuous intravenous infusion on day 1, and S-1 was given orally for 2 weeks, followed by a 1-week rest period. This 3-week regimen was regarded as 1 course. The RR was 67.0 % (95 % CI, 52.1 to 79.1 %), and the PFS was 12.4 months (95 % CI, 10.0 to 14.7 months). Kato et al. [10] evaluated S-1 plus irinotecan combined with bevacizumab as first- and second-line treatment. Bevacizumab (7.5 mg/kg) and irinotecan (150 mg/m^2^) were given as a continuous intravenous infusion on day 1, and S-1 was given orally for 2 weeks, starting on day 3, every 3 weeks, defined as 1 course. The RR was 72.0 % (95 % CI, 50.6 to 86.2 %), and the PFS was 11.5 months (95 % CI, 10.4 to 19.8 months).

The results of these studies demonstrated that a PFS of 10 months can be expected with either a 4-week course (IRIS/bevacizumab) or a 3-week course (SIRB) of S-1 plus irinotecan combined with bevacizumab. Although the dose of irinotecan in these regimens was 150 mg/m^2^ every 3 weeks, which is lower than the dose in FOLFIRI (270 mg/m^2^ every 3 weeks), we considered this dose of irinotecan to be appropriate for combination therapy with S-1, irinotecan, and bevacizumab.

All studies of combination therapy with S-1, irinotecan, and bevacizumab have confirmed that this regimen is well tolerated: the incidence of grade 3 or higher neutropenia was 14 to 27 %, and the incidence of grade 3 or higher diarrhea was 7 to 17 %. Although some patients had elevations of blood pressure or thrombosis (i.e., adverse effects most likely attributed to bevacizumab), concurrent treatment with bevacizumab did not increase S-1- or irinotecan-related toxicity. The results of these 3 phase II studies suggested that a combination of S-1, irinotecan, and bevacizumab (S-1/irinotecan/bevacizumab) is comparable to FOLFOX/bevacizumab and CapeOX/bevacizumab, currently used as standard regimens for metastatic colorectal cancer, in terms of PFS. S-1/irinotecan/bevacizumab can be given as a 3-week regimen (SIRB) or a 4-week regimen (IRIS/bevacizumab).

Standard regimens of FOLFOX/bevacizumab and CapeOX/bevacizumab can cause neurotoxicity, potentially disrupting treatment. In the NO16966 study, the PFS was 9.4 months in the FOLFOX/bevacizumab and CapeOX/bevacizumab groups combined, as compared with a time to treatment failure (TTF) of only 6.9 months. The reason for treatment withdrawal was disease progression in 29 % of the FOLFOX/bevacizumab and CapeOX/bevacizumab groups combined and adverse events in 32 %. The main reasons for treatment withdrawal were not adverse events caused by bevacizumab, but neuropathy and other adverse events caused by chemotherapy. The use of FOLFOX/CapeOX as first-line treatment is associated with a higher total dose of oxaliplatin than second-line treatment, resulting in a higher incidence and severity of neuropathy [11]. In addition, the use of these regimens as second-line treatment would prolong exposure to neurotoxicity during the overall survival period. Therefore, irinotecan-based chemotherapy should be used as first-line treatment from the viewpoint of toxicity and patients’ QOL. At present, oxaliplatin-based regimens are mainly used for postoperative adjuvant chemotherapy. In patients who have recurrence during or immediately after oxaliplatin-based chemotherapy, the cumulative neurotoxic effects of oxaliplatin may persist [12]. Therefore, the establishment and evaluation of S-1-containing irinotecan-based regimens for first-line treatment are expected to become more important.

On the basis of these findings, the primary endpoint of this study is to validate the non-inferiority of combination therapy with S-1/irinotecan/bevacizumab (SIRB or IRIS/bevacizumab) to oxaliplatin-based standard treatment (mFOLFOX6/bevacizumab or CapeOX/bevacizumab) in terms of PFS and to assess superiority if non-inferiority is demonstrated. Whether it is possible to quantitatively predict differences in response to irinotecan-based regimens and oxaliplatin-based regimens will also be investigated, with the ultimate goal of improving patients’ QOL, healthcare cost-performance, and individualized therapy of colorectal cancer.

## Methods and study design

### Study design

**Fig. 1:**
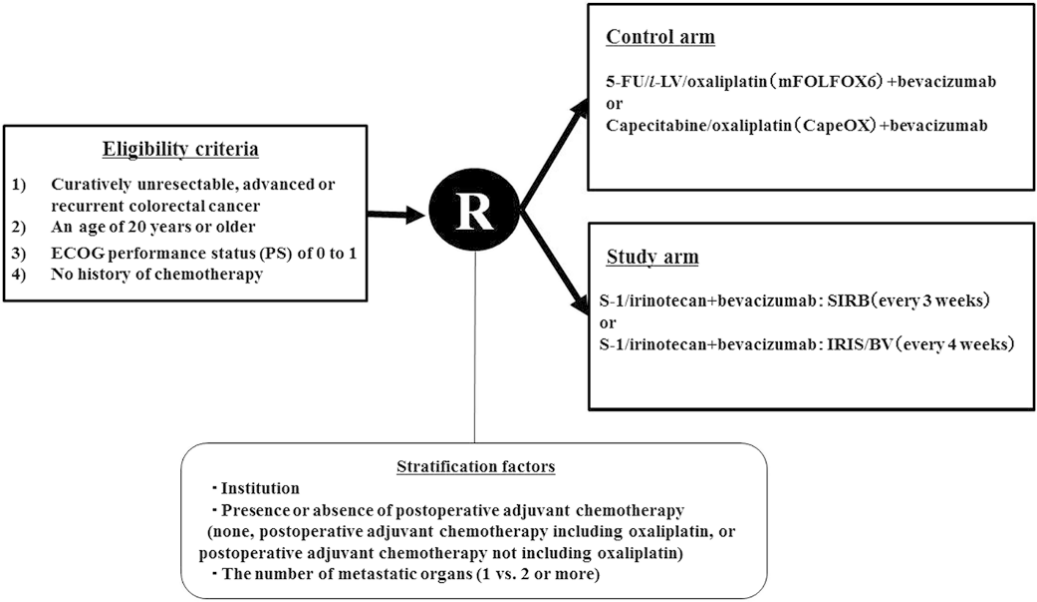
Study schema

This is a multicenter, randomized, open-label, controlled phase III study of patients who receive first-line treatment for metastatic colorectal cancer. The control group will receive either mFOLFOX6/bevacizumab or CapeOX/bevacizumab, preassigned according to institution (Fig. 1). The study group will receive either SIRB or IRIS/bevacizumab. Patients will be randomly assigned to either the control group or study group. Randomization will be performed using a minimization method with the following stratification factors: institution and the presence or absence of postoperative adjuvant chemotherapy (none, postoperative adjuvant chemotherapy including oxaliplatin, or postoperative adjuvant chemotherapy not including oxaliplatin), and the number of metastatic organs (1 vs. 2 or more).

### Purpose of the study

This study is designed to validate the non-inferiority of combination therapy with S-1/irinotecan/bevacizumab (SIRB or IRIS/bevacizumab) to oxaliplatin-based standard treatment (mFOLFOX6/bevacizumab or CapeOX/bevacizumab) in terms of PFS, the primary endpoint, in patients with metastatic colorectal cancer who had not received chemotherapy. Secondary endpoints are overall survival (OS), response rate (RR), TTF, relative dose intensity (RDI), the incidence and severity of adverse events, QOL, quality-adjusted life years (QALY), health care costs, and relations between biomarkers and treatment response (translational research, TR).

### Enrollment and allocation

**Fig. 2:**
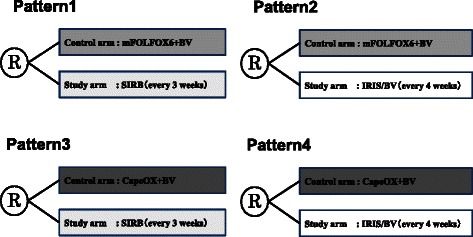
Pattern of treatment at each institution

Eligible patients will be randomly assigned to treatment by means of a Web-based system managed by Medical Toukei Corporation (Tokyo, Japan), independently from the sponsor and participating institutions. Patients will be randomly assigned to the control group (FOLFOX/bevacizumab or CapeOX/bevacizumab) or study group (SIRB or IRIS/bevacizumab) according to the pattern at each institution (Fig. 2).

The main inclusion criteria are as follows: a histologically confirmed diagnosis of colorectal adenocarcinoma; curatively unresectable, advanced or recurrent colorectal cancer; an age of 20 years or older at enrollment; an Eastern Cooperative Oncology Group performance status (PS) of 0 or 1; confirmed presence of assessable lesions on objective examinations such as computed tomography, magnetic resonance imaging, and radiography (measurable lesions not required); no history of chemotherapy or radiotherapy (However, patients who had received postoperative adjuvant chemotherapy were eligible provided that at least 180 days had elapsed since the final day of treatment.); adequate oral intake; a leucocyte count, ≥3000/μL; a neutrophil count, ≥1500/μL; a platelet count, ≥100 × 10^3^/μL; a hemoglobin level, ≥9.0 g/dL; a total bilirubin level, ≤1.5 mg/dL; an aspartate aminotransferase(AST) level, ≤100 IU/L(≤150 IU/L in patients with hepatic metastasis); an alanine aminotransferase(ALT) level, ≤100 IU/L(≤150 IU/L in patients with hepatic metastasis); a serum creatinine level, ≤1.2 mg/dL; a creatinine clearance, ≥60 mL/min; an urinary protein level, ≤1+; an expected survival of at least 3 months; and written informed consent from the patient.

The main exclusion criteria are as follows: a history of serious drug sensitivity; women who are pregnant or may become pregnant; men who want their partner to become pregnant; active infectious disease; serious complications; marked electrocardiographic abnormalities or clinically problematic heart disease; gastrointestinal ulcer or bleeding; sensory neuropathy; serious diarrhea; ascites or pleural effusion requiring treatment; gastrointestinal obstruction; symptomatic peritoneal metastasis; brain metastasis or brain metastasis suspected on the basis of clinical symptoms; a history of gastrointestinal perforation within 6 months before enrollment (Patients with gastrointestinal perforation due to obstructive colorectal cancer are eligible.); a history of hemoptysis; a history and complications of embolism, cerebral infarction (Patients with asymptomatic lacunar infarction are eligible.), lung infarction, or interstitial pneumonia; surgery performed within 28 days before enrollment; congenital bleeding disorders or coagulation abnormalities; treatment with anticoagulants; blood transfusion required within 14 days before enrollment; another active cancer with a disease-free interval of less than 5 years; continuous systemic treatment with steroids; positivity for hepatitis B surface antigen; and patients considered unsuitable for participation in the study by their attending physicians.

### Protocol treatment

#### Control arm

##### mFOLFOX6/bevacizumab regimen

In patients assigned to the mFOLFOX6/bevacizumab regimen, bevacizumab (5 mg/kg) is given as a continuous intravenous infusion over the course of 30 to 90 min on day 1, followed by a continuous intravenous infusion of oxaliplatin (85 mg/m^2^) plus *l*-leucovorin (200 mg/m^2^) given over the course of 2 h. Immediately after completing the infusion of oxaliplatin and *l*-leucovorin, 5-fluorouracil (400 mg/m^2^) is given by rapid intravenous injection, followed by a continuous intravenous infusion of 5-fluorouracil (2400 mg/m^2^), delivered with the use of an infusion pump over the course of 46 h. This 2-week course of treatment is repeated.

##### CapeOX/bevacizumab regimen

In patients assigned to the CapeOX/bevacizumab regimen, bevacizumab (7.5 mg/kg) is given as a continuous intravenous infusion over the course of 30 to 90 min on day 1, followed by a continuous intravenous infusion of oxaliplatin (130 mg/m^2^) over the course of 2 h. The dose of capecitabine will be assigned according to body surface area (BSA) as follows: BSA <1.36 m^2^, 2400 mg/day; BSA ≥1.36 m^2^ to <1.66 m^2^, 3000 mg/day; BSA ≥1.66 m^2^ to <1.96 m^2^, 3600 mg/day; and BSA ≥1.96 m^2^, 4200 mg/day. Capecitabine is given orally twice daily after breakfast and dinner, starting after dinner on day 1 to after breakfast on day 15, followed by 14-day rest period. This 28-day regimen is defined as 1 course of treatment and is repeated.

Treatment with the mFOLFOX6/bevacizumab regimen and CapeOX/bevacizumab regimen will be begun in patients who meet all of the following criteria: leucocyte count, ≥3000/μL; neutrophil count, ≥1500/μL; platelet count, ≥100 × 10^3^/μL; AST and ALT, ≤100 IU/L (≤150 IU/L in patients with hepatic metastasis); total bilirubin level, ≤1.5 mg/dL; serum creatinine level, ≤1.2 mg/dL; no fever of ≥38 °C suggesting infection; and ≤ grade 1 diarrhea and mucositis/stomatitis.

Patients who have ≤ grade 2 neuropathy (sensory) will receive 5-fluorouracil/*l*-leucovorin/oxaliplatin plus bevacizumab or CapeOX/bevacizumab. Patients with grade 3 neuropathy (sensory) will be given 5-fluorouracil/*l*–leucovorin/bevacizumab or capecitabine/bevacizumab. In patients with ≤ grade 1 hand-foot syndrome, treatment will be started with the CapeOX/bevacizumab regimen.

Dose-reduction criteria for the mFOLFOX6/bevacizumab regimen and CapeOX/bevacizumab regimen are as follows. In patients who have a leucocyte count of <1000/μL, a neutrophil count of <500/μL, or a neutrophil count of <1500/μL on the day scheduled for the next course of treatment to begin, and in those who have ≥ grade 3 febrile neutropenia, a platelet count of <50 × 10^3^/μL, or AST and ALT levels of ≥200 IU/L, the dose of 5-fluorouracil or the doses of capecitabine and oxaliplatin are decreased by 1 level. In patients who have ≥ grade 3 diarrhea, the dose of 5-fluorouracil is decreased by 1 dose level. In patients who have ≥ grade 3 diarrhea and hand-foot syndrome or 2 or more episodes of grade 2 diarrhea and hand-foot syndrome, the dose of capecitabine is decreased by 1 level. In patients who have a platelet count of ≥50 × 10^3^/μL or <75 × 10^3^/μL, the dose of only oxaliplatin is decreased by 1 level.

#### Study arms

##### SIRB regimen

The SIRB regimen consists of bevacizumab (7.5 mg/kg) given as a continuous intravenous infusion over the course of 30 to 90 min on day 1, followed by a continuous intravenous infusion of irinotecan (150 mg/m^2^) given over the course of ≥90 min. The dose of S-1 will be determined according to BSA as follows: BSA <1.25 m^2^, 80 mg/day; BSA ≥1.25 m^2^ to <1.5 m^2^, 100 mg/day; and BSA ≥1.5 m^2^, 120 mg/day. S-1 is given orally twice daily after breakfast and dinner, starting from after dinner on day 1 to after breakfast on day 15, followed by 7-day rest. This 21-day regimen is defined as 1 course of treatment and will be repeated.

##### IRIS/bevacizumab regimen

The IRIS/bevacizumab regimen consists of bevacizumab (5 mg/kg) given as a continuous intravenous infusion over the course of 30 to 90 min on day 1 and day 15, followed by a continuous intravenous infusion of irinotecan (100 mg/m^2^), given over the course of ≥90 min. The dose of S-1 will be determined according to BSA as follows: BSA <1.25 m^2^, 80 mg/day; BSA ≥1.25 m^2^ to <1.5 m^2^, 100 mg/day; and BSA ≥1.5 m^2^, 120 mg/day. S-1 is given orally twice daily after breakfast and dinner, starting from after dinner on day 1 to after breakfast on day 15, followed by a 14-day rest. This 28-day regimen is defined as 1 course of treatment and will be repeated.

Treatment with the SIRB regimen and IRIS/bevacizumab regimen is begun in patients who meet all of the following criteria: leucocyte count, ≥3000/μL; neutrophil count, ≥1500/μL; platelet count, ≥100 × 10^3^/μL; AST and ALT, ≤100 IU/L (≤150 IU/L in patients with hepatic metastasis); total bilirubin level, ≤1.5 mg/dL; serum creatinine level, ≤1.2 mg/dL; no fever of ≥38 °C suggesting infection; and ≤ grade 1 diarrhea and mucositis/stomatitis.

Dose-reduction criteria for the SIRB regimen and IRIS/bevacizumab regimen are as follows. In patients who have a leucocyte count of <1000/μL, a neutrophil count of <500/μL, or a neutrophil count of <1500/μL on the day scheduled for the next course of treatment to begin, and in those who have ≥ grade 3 febrile neutropenia, a platelet count of <50 × 10^3^/μL, AST and ALT levels of ≥200 IU/L, or ≥ grade 3 diarrhea, the doses of S-1 and irinotecan are each decreased by 1 level. In patients who have a platelet count of ≥50 × 10^3^/μL or <75 × 10^3^/μL, the dose of only oxaliplatin is decreased by 1 level. In patients who have a serum creatinine level of ≥1.5 mg/dL or ≥ grade 3 mucositis or stomatitis, the dose of S-1 is decreased by 1 level.

##### Withdrawal criteria

Treatment in each group is repeated until patients meet the treatment withdrawal criteria. The criteria for the withdrawal of the study treatment are as follows: clear exacerbation of the primary disease; the subject requests discontinuation of the study treatment; the attending physician judges that continuation of the study treatment is precluded by the development of adverse events; it becomes difficult for the subject to continuously consult the attending physician because of changes in residence or hospitals or because of other commitments; surgery is performed or considered feasible because of tumor shrinkage; patients are found to be ineligible after enrollment; or the attending physician judges that continuation of the study treatment is precluded by other reasons.

### Evaluation of treatment delivery and adverse events

#### Treatment delivery

Drug administration status such as dosage and the treatment period will be recorded in the patients’ case report forms. Whether treatment is temporarily withheld or the dosage reduced and the reasons for such modifications will also be recorded. Relative dose intensity (RDI) will be calculated on the basis of these findings.

#### Safety profile

In this study, adverse events are followed up for 30 days after the final dose of the study treatment or until the start of subsequent therapy. Observed adverse events are evaluated according to the Common Terminology Criteria for Adverse Events, version 4.0 (CTCAE, v4.0).

### Statistical background

#### Definition of primary endpoint

The primary endpoint of the study is PFS. PFS is defined as the period from the date of enrollment to the date of disease progression or of death from any cause, whichever comes first. Data are censored on the date of surgery for patients who undergo surgical resection of tumors that respond to the protocol treatment and become resectable. In patients with confirmed disease progression (recurrence in patients who undergo R0 resection) after surgery, the confirmation date is considered to indicate an event.

#### Definition of analysis sets

The full analysis set (FAS) is defined as all enrolled patients. However, patients with serious study protocol violations (i.e., informed consent not obtained or serious violations of the study procedures) will be excluded. The per protocol set (PPS) is defined as the patients remaining after exclusion of patients with serious violations of factors such the eligibility criteria, exclusion criteria, or contraindicated drugs or therapy. The safety analysis set (SAS) is defined as all enrolled patients after excluding those who did not receive any of the study treatment.

#### Definition of target sample size

On the basis of the results of the NO16966 study [2, 3], the median PFS is estimated to be 11 months for oxaliplatin-based regimens such as FOLFOX/bevacizumab and CapeOX/bevacizumab and 12 months for S-1/irinotecan/bevacizumab (hazard ratio, 0.917). Given that the permissible limit for the hazard ratio is 1.25, with a statistical power of 85 %, an alpha level of 0.025 (one-sided), an enrollment period of 36 months, and a follow-up period of 18 months for the primary endpoint of PFS, 434 patients are estimated to be required (required number of events, 374). To compensate for ineligible patients, the target number of patients is set at 450. If the non-inferiority is demonstrated in the study, superiority will be tested. (The enrollment of 434 patients is estimated to provide 78 % statistical power to detect a difference between the groups with a hazard ratio of 0.75).

#### Analysis plan

All evaluations of treatment effectiveness will be performed in the FAS. As a reference, effectiveness will also be analyzed in the PPS. Survival times (PFS, OS, and TTF) are estimated by the Kaplan-Meier method. Two-sided 95 % confidence intervals of the hazard ratios in the assigned study groups are calculated by using a Cox proportional-hazards model adjusted for stratification factors other than institution and including only the study group as a covariate. The upper limit of the confidence interval will be confirmed to be below 1.25. Superiority in terms of PFS will be tested using a stratified log-rank test adjusted for stratification factors other than institution.

Response rates with confidence intervals will be calculated according to the assigned group for patients who have measurable lesions. Differences in response rates between assigned treatment groups will also be calculated along with the respective confidence intervals. The best overall response rates will be calculated according to the assigned group. A chi-square test (Fisher’s exact test as needed) will be used to compare the assigned groups.

To assess treatment achievement status, the RDI of each drug used in each treatment group will be calculated, and summary statistics, including the medians and means, will be reported. To evaluate safety, the incidences of adverse events along with the confidence intervals and grades will be calculated for each assigned group. Chi-square tests (Fisher’s exact tests as required) will be used to compare the assigned treatment groups.

### Quality of life (QOL)

QOL variables will be evaluated with the use of the Functional Assessment of Cancer Therapy–Colorectal (FACT–C) [13], neurotoxicity subscale of the FACT/Gynecology Oncology Group-Neurotoxicity (FACT/GOG-Ntx) [14], QOL Questionnaire for Cancer Patients Treated with Anticancer Drugs therapy (QOL-ACD) [15], and the Japanese version of the EuroQol Group 5-Dimension Self-Report Questionnaire (EQ-5D) [16]. Quality-adjusted life-years (QALY) will be calculated on the basis of OS and the utility values calculated from the results obtained for the 5 dimensions of EQ-5D.

QOL surveys will be performed before treatment, 16 weeks after treatment begins, 24 weeks after treatment begins, and at the time of treatment withdrawal. After withdrawal of the study treatment, only the FACT/GOG-Ntx assessment will be performed 12 weeks and 24 weeks after the completion of treatment.

### Cost-effectiveness analysis

Medical care costs required for the study treatment and supportive care will be calculated for each course of treatment per patient and compared between the control group and the study group.

During the period from enrollment to completion of the study treatment, data on the dose levels of each drug, the number of administered doses, catheter placement, port placement, methods for intravenous infusion management, methods for supportive care, and hospital admissions will be collected and recorded in each patient’s case report form and used to calculate the cost per course of treatment.

For patients treated at hospitals that cooperated in this survey by providing the details of invoices for healthcare remuneration (claim data), lifetime total medical care costs will be calculated. For the period from initiation of the study treatment until death, medical care costs according to procedure and total medical care costs will be calculated for each subject on a lifetime basis and during the study treatment, and total medical care costs will be compared between the control group and the study group. In addition, after the study treatment begins, the attending physician will fill in a patient questionnaire concerning the use of nursing and long-term care services, informal care provided by family members, and disease-related changes in the employment status of the patients and their family members. These data will be used to compare non-medical care costs between the control group and the study group.

The aforementioned data on medical care costs and non-medical care costs and the QALY data will be used to calculate incremental cost-effectiveness ratios in the control group and the study group.

### Translational research (TR)

Colorectal cancer can be broadly classified into 4 subtypes according to two elements. Takahashi et al. reported that the effectiveness of irinotecan-based regimens and oxaliplatin-based regimens might differ according to subtype. For patients who participate in this study and consent to providing tissue specimens, correlations of biomarkers in histopathological specimens with outcomes and with the clinical effectiveness of chemotherapy in this study will be evaluated to determine whether the effectiveness of irinotecan-based regimens and oxaliplatin-based regimens can be predicted on the basis of subtype classifications derived from comprehensive gene analysis. New biomarkers will also be investigated.

The KRAS, NRAS, BRAF, and PIK3CA genes, CACNA1G, IGF2, NEUROG1, RUNX3, SOCS1, and MLH1 as methylation markers for CpG island methylator phenotype (CIMP), and other genes useful for the evaluation of CIMP will be analyzed. Immunohistochemical analyses of MLH1, MSH2, PMS2, MSH6, and PTEN will be performed.

### Ethical matters

This study will be conducted in accordance with the Declaration of Helsinki and the ethical principles of “Ethical Guidelines for Clinical Research” in Japan to maximally ensure the human rights, welfare, and safety of the subjects. Translational research variables evaluated in this study will be evaluated according to “Ethical Guidelines for Human Genome and Genetic Sequencing Research.” Ethical approval has been obtained at all participating centers Additional file 1: Table S1).

### Decision criteria

Decision criteria will be determined by the steering committee as follows. If S-1/irinotecan/bevacizumab is demonstrated to be non-inferior in terms of the primary endpoint of PFS and is superior to mFOLFOX6/bevacizumab or CapeOX/bevacizumab with respect to one or more of the secondary endpoints of QOL, cost performance, and the incidence of peripheral neuropathy, S-1/irinotecan/bevacizumab will be comprehensively evaluated to be positioned as a standard first-line treatment for metastatic colorectal cancer, taking into account the incidence and severity of adverse events as well as other secondary endpoints.

If superiority is demonstrated in terms of PFS and the incidence of serious adverse events is within acceptable limits, S-1/irinotecan/bevacizumab will be positioned as a standard treatment for metastatic colorectal cancer from the time of this study.

## Discussion

The present study is designed to comprehensively analyze 2 regimens each in the control group and the study group. Both mFOLFOX6/bevacizumab and CapeOX/bevacizumab, used in the control group, are standard treatments widely used in Japan. The results of phase II studies of IRIS/bevacizumab and SIRB, used in the study group, suggest that the efficacy and side effect profiles of these regimens are generally similar. Evidence from Phase III studies has been obtained for IRIS/bevacizumab, but only the results of phase II studies are available for SIRB. However, the SIRB regimen has the advantage that patients are required to come to the hospital only once every 3 weeks. Because each regimen has advantages, 2 regimens are included in the study arm of the present study.

At present, both oxaliplatin-based regimens and irinotecan-based regimens are recommended as first-line standard treatment for advanced or recurrent colorectal cancer. In general clinical practice in Japan, oxaliplatin-based regimens are usually used initially. However, given the peripheral neuropathy associated with oxaliplatin, first-line treatment with irinotecan-based regimens might be better with respect to toxicity. In our study, combining the oral preparation S-1 with irinotecan instead of using FOLFIRI, a regimen combining intravenous 5-fluorouracil/*l*-leucovorin with irinotecan, is expected to reduce drug-related costs and improve the patients’ QOL because the use of a portable infusion pump for 2 days is not required.

In our study, the results of QOL surveys, translational research, and cost effectiveness analysis are secondary endpoints. We believe that our study is very meaningful from the perspective of comparative effectiveness research.

## Additional file

Additional file 1: **Table S1.** Ethic committee. (PPTX 81 kb)

## Abbreviations

ALT: Alanine aminotransferase
AST: Aspartate aminotransferase
CTCAE v4.0: Common Terminology Criteria for Adverse Events v4.0
FAS: Full analysis set
OS: Overall survival
PFS: Progression-free survival
PPS: Per protocol set
PS(ECOG): Performance status(Eastern Cooperative Oncology Group)
QOL: Quality of life
QALY: Quality-adjusted life years
RDI: Relative dose intensity
RECIST: Response evaluation criteria in solid tumors
RR: Response rate
TTF: Time to treatment failure
TR: Translational research
