# Supplementary material for: Study protocol of the TRICOLORE trial: a randomized phase III study of oxaliplatin-based chemotherapy versus combination chemotherapy with S-1, irinotecan, and bevacizumab as first-line therapy for metastatic colorectal cancer
Source: BMC Cancer. 2015 Sep 9;15:626. doi: 10.1186/s12885-015-1630-1 (PMC4565014; doi:10.1186/s12885-015-1630-1)
Supplement: Additional file 1: — Table S1. Ethic committee. (PPTX 81 kb) [file 12885_2015_1630_MOESM1_ESM.pptx]

## Slide 1
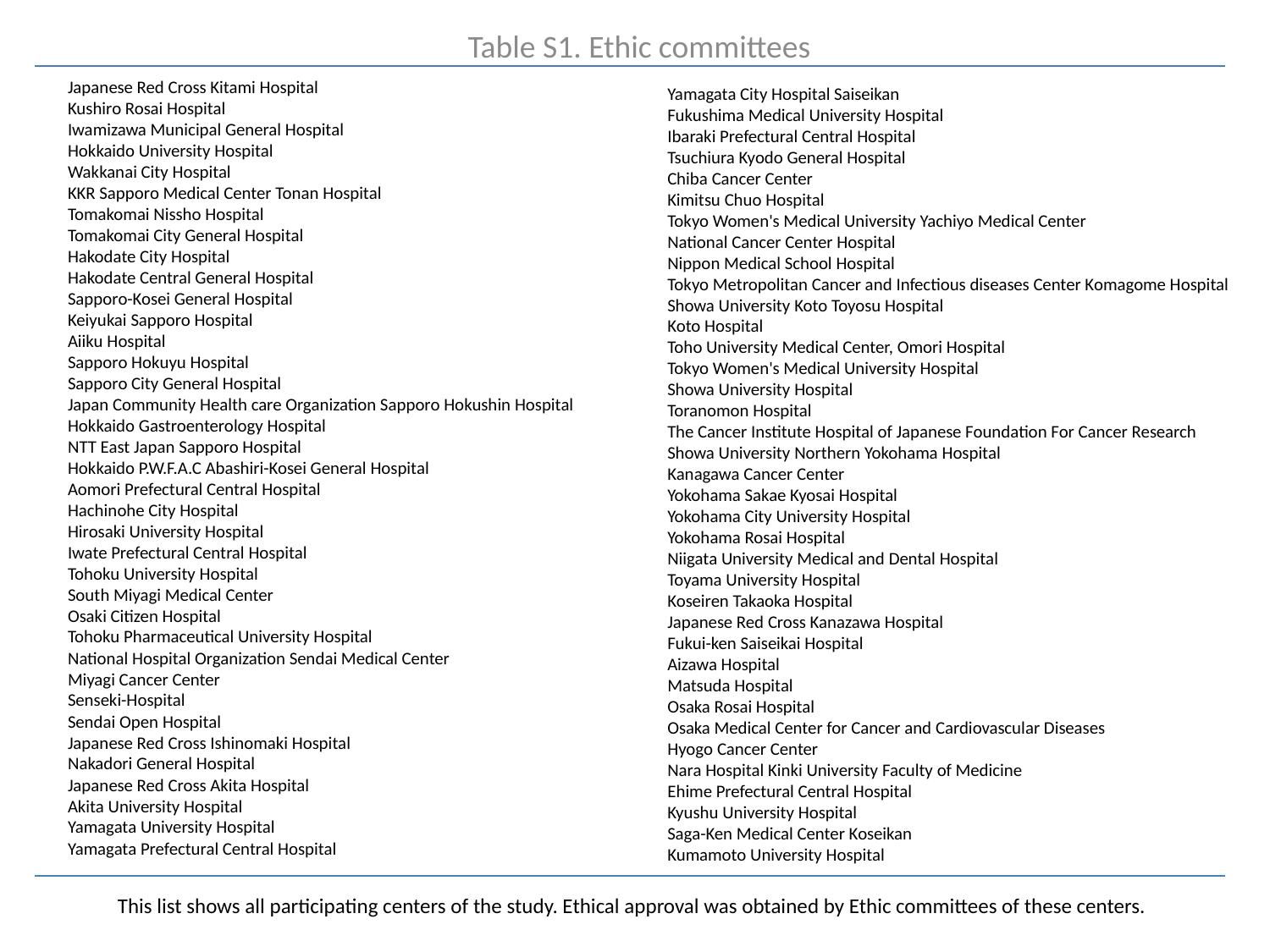

Table S1. Ethic committees
Japanese Red Cross Kitami Hospital
Kushiro Rosai Hospital
Iwamizawa Municipal General Hospital
Hokkaido University Hospital
Wakkanai City Hospital
KKR Sapporo Medical Center Tonan Hospital
Tomakomai Nissho Hospital
Tomakomai City General Hospital
Hakodate City Hospital
Hakodate Central General Hospital
Sapporo-Kosei General Hospital
Keiyukai Sapporo Hospital
Aiiku Hospital
Sapporo Hokuyu Hospital
Sapporo City General Hospital
Japan Community Health care Organization Sapporo Hokushin Hospital
Hokkaido Gastroenterology Hospital
NTT East Japan Sapporo Hospital
Hokkaido P.W.F.A.C Abashiri-Kosei General Hospital
Aomori Prefectural Central Hospital
Hachinohe City Hospital
Hirosaki University Hospital
Iwate Prefectural Central Hospital
Tohoku University Hospital
South Miyagi Medical Center
Osaki Citizen Hospital
Tohoku Pharmaceutical University Hospital
National Hospital Organization Sendai Medical Center
Miyagi Cancer Center
Senseki-Hospital
Sendai Open Hospital
Japanese Red Cross Ishinomaki Hospital
Nakadori General Hospital
Japanese Red Cross Akita Hospital
Akita University Hospital
Yamagata University Hospital
Yamagata Prefectural Central Hospital
Yamagata City Hospital Saiseikan
Fukushima Medical University Hospital
Ibaraki Prefectural Central Hospital
Tsuchiura Kyodo General Hospital
Chiba Cancer Center
Kimitsu Chuo Hospital
Tokyo Women's Medical University Yachiyo Medical Center
National Cancer Center Hospital
Nippon Medical School Hospital
Tokyo Metropolitan Cancer and Infectious diseases Center Komagome Hospital
Showa University Koto Toyosu Hospital
Koto Hospital
Toho University Medical Center, Omori Hospital
Tokyo Women's Medical University Hospital
Showa University Hospital
Toranomon Hospital
The Cancer Institute Hospital of Japanese Foundation For Cancer Research
Showa University Northern Yokohama Hospital
Kanagawa Cancer Center
Yokohama Sakae Kyosai Hospital
Yokohama City University Hospital
Yokohama Rosai Hospital
Niigata University Medical and Dental Hospital
Toyama University Hospital
Koseiren Takaoka Hospital
Japanese Red Cross Kanazawa Hospital
Fukui-ken Saiseikai Hospital
Aizawa Hospital
Matsuda Hospital
Osaka Rosai Hospital
Osaka Medical Center for Cancer and Cardiovascular Diseases
Hyogo Cancer Center
Nara Hospital Kinki University Faculty of Medicine
Ehime Prefectural Central Hospital
Kyushu University Hospital
Saga-Ken Medical Center Koseikan
Kumamoto University Hospital
This list shows all participating centers of the study. Ethical approval was obtained by Ethic committees of these centers.
